# Intensive trapping of blood-fed *Anopheles darlingi* in Amazonian Peru reveals unexpectedly high proportions of avian blood-meals

**DOI:** 10.1371/journal.pntd.0005337

**Published:** 2017-02-23

**Authors:** Marta Moreno, Marlon P. Saavedra, Sara A. Bickersmith, Catharine Prussing, Adrian Michalski, Carlos Tong Rios, Joseph M. Vinetz, Jan E. Conn

**Affiliations:** 1 Division of Infectious Diseases, Department of Medicine, University of California San Diego, La Jolla, California, United States of America; 2 Laboratorio ICEMR-Amazonia, Laboratorios de Investigacion y Desarrollo, Facultad de Ciencias y Filosofia, Universidad Peruana Cayetano Heredia, Lima, Peru; 3 Wadsworth Center, New York State Department of Health, Albany, NY, United States of America; 4 Department of Biomedical Sciences, School of Public Health, State University of New York-Albany, NY, United States of America; Fundaçao Oswaldo Cruz, BRAZIL

## Abstract

*Anopheles darlingi*, the main malaria vector in the Neotropics, has been considered to be highly anthropophilic. However, many behavioral aspects of this species remain unknown, such as the range of blood-meal sources. Barrier screens were used to collect resting *Anopheles darlingi* mosquitoes from 2013 to 2015 in three riverine localities (Lupuna, Cahuide and Santa Emilia) in Amazonian Peru. Overall, the Human Blood Index (HBI) ranged from 0.58–0.87, with no significant variation among years or sites. Blood-meal analysis revealed that humans are the most common blood source, followed by avian hosts (Galliformes-chickens and turkeys), and human/Galliforme mixed-meals. The Forage Ratio and Selection Index both show a strong preference for Galliformes over humans in blood-fed mosquitoes. Our data show that 30% of *An*. *darlingi* fed on more than one host, including combinations of dogs, pigs, goats and rats. There appears to be a pattern of host choice in *An*. *darlingi*, with varying proportions of mosquitoes feeding only on humans, only on Galliformes and some taking mixed-meals of blood (human plus Galliforme), which was detected in the three sites in different years, indicating that there could be a structure to these populations based on blood-feeding preferences. Mosquito age, estimated in two localities, Lupuna and Cahuide, ranged widely between sites and years. This variation may reflect the range of local environmental factors that influence longevity or possibly potential changes in the ability of the mosquito to transmit the parasite. Of 6,204 resting *An*. *darlingi* tested for *Plasmodium* infection, 0.42% were infected with *P*. *vivax*. This study provides evidence for the first time of the usefulness of barrier screens for the collection of blood-fed resting mosquitoes to calculate the Human Blood Index (HBI) and other blood-meal sources in a neotropical malaria endemic setting.

## Author summary

*Anopheles darlingi* is the major malaria vector in the Amazon. This species has been commonly described as highly anthropophilic throughout its geographic range, although little is known about its feeding preferences. Scant information is available regarding the origin of *An*. *darlingi* blood-meals. In the context of malaria elimination programs, the Human Blood Index (HBI) may provide crucial information regarding mosquito-human contact related to transmission dynamics. Additionally, collection of resting *An*. *darlingi* is challenging, mainly because the resting behavior of this species has not been well characterized. Our study, conducted from 2013–2015 in three localities in Loreto Department in the Peruvian Amazon, showed for the first time the efficacy of the barrier screen methodology for collecting recently blood-fed *An*. *darlingi* in a neotropical setting for the purpose of identifying the source of their blood-meals. Our data show that *An*. *darlingi* feeds on humans, Galliformes, dogs, pigs and goats, and that 30% of the mosquitoes fed on more than one type of host. Despite this opportunistic feeding behavior, however, *An*. *darlingi* is primarily anthropophilic. We hypothesize that mosquito population structure is associated with feeding preferences, which may affect the pattern of malaria transmission in the area.

## Introduction

The Human Blood Index (HBI), formerly known as the anthropophilic index or human blood ratio, is the proportion of recently-fed mosquitoes, usually vector species that have taken a human blood-meal [1]. This index is a very important component of the formulae used to determine vectorial capacity and varies depending on mosquito species, collection area and season or time of collection [2]. From an epidemiological standpoint, it is crucial to be able to accurately identify mosquito blood-meals for studies of transmission dynamics of viral and parasitic pathogens [3]. For example, in Equatorial Guinea, the calculation of this index before and after indoor interventions to reduce malaria did not detect any mosquito behavioral differences, and researchers concluded that control strategies in this region were ineffective [4]. In Central Kenya, anthropophily decreased in *An*. *gambiae* after the introduction of long lasting insecticide nets (LLINs) and zooprophylaxis [5]. However, in southern Zambia, after two years of LLIN intervention, the main vector, *Anopheles arabiensis*, remained highly anthropophilic [6]. In Tanzania the HBI showed a change in the main blood-source in *An*. *arabiensis* but not in *An*. *funestus* after the use of spatial repellent coils [7].

Another index to quantify host selection patterns is the incidence of multiple blood-meals from the same host species (cryptic) or from two or more different host species (patent) [8]. Evidence that malarial mosquitoes take partial blood-meals from multiple hosts may be interpreted as interrupted blood-feedings that could increase the probability of both acquiring and transmitting *Plasmodium* [9]. On the other hand, Burkot and colleagues [10] contend that fewer gametocytes would be ingested per meal, resulting in lower mosquito infection rates.

*Anopheles darlingi*, the primary regional malaria vector in the Amazon Basin, is anthropophilic in the Iquitos region [11], although both human biting rate (HBR) and entomological inoculation rate (EIR) vary widely [12] depending on the setting [13–15]. The *An*. *darlingi* feeding site in this region is exophagic and/or endophagic, depending on local circumstances (e.g., vegetation cover, type of house) and host availability [11, 12, 14,15].

In 2015, Loreto Department reported 95% of the total malaria cases in Peru (59,349 of 62,220 total) with *Plasmodium vivax* as the most prevalent human parasite followed by *P*. *falciparum*, with 46,924 and 12,425 cases, respectively [16]. Parker and collaborators [13] demonstrated that high HBR, EIR, and infectivity of *An*. *darlingi* are a signature of remote riverine malaria hot spots and hyperendemicity in certain areas of the Peruvian Amazon, upending previous notions that transmission is hypoendemic throughout the peri-Iquitos region [11,12]. Recent studies also detected very high seasonal HBR and moderate EIR in the peri-Iquitos region [14, 15]. Most malaria cases occur during the rainy season, from December to June [17] and a correlation was detected between *An*. *darlingi* abundance and peak river levels, but there was no significant correlation between river level and malaria case numbers [12, 14, 15]. In this last study, mosquitoes positive for *Plasmodium* were collected in peridomestic areas within approximately 10 m of the main house entrance, (a caveat being that very few *An*. *darlingi* were found indoors despite extensive searching), suggesting that most malaria is transmitted exophagically, where humans have little protection against mosquito bites.

Despite being the dominant malaria vector in Amazonia, few studies have documented the blood-meal sources for *An*. *darlingi*. In Amapá state, Amazonian Brazil, an ELISA analysis found that 13.1% of blood-meals were human; most resting *An*. *darlingi* had fed on cattle, pigs and dogs [18]. Notwithstanding the relatively low level of HBI, these communities are endemic for malaria, and *An*. *darlingi* is considered to be the most effective local vector [19]. In Peru, no studies have been published on the identity of *An*. *darlingi* blood-meals, but potential non-human hosts in rural residences near Iquitos include common peridomestic animals, dogs and chickens, and several potential wild mammalian hosts [12].

Although resting mosquitoes are optimal for calculating HBI, adequate sample sizes can be difficult to obtain in some habitats [18–20]. Little information exists on host preference and resting behavior of *An*. *darlingi*. The location of resting sites of *An*. *darlingi* could be useful for focal vector control if such mosquitoes are clustered non-randomly in the landscape. The development of barrier screens as a method for collecting anophelines outdoors has been tested successfully in the South East Pacific [20] and recently in southern Zambia [21].

This study was designed to address the following questions regarding *An*. *darlingi* feeding behavior in the Peruvian Amazon: i) are barrier screens a useful tool to collect resting blood-fed *An*. *darlingi* in the area; ii) what is the degree of anthropophily (HBI) in *An*. *darlingi* in contrast to more opportunistic behavior; iii) what is the influence of available host biomass and iv) is there evidence of seasonal age-structure in *An*. *darlingi*.

## Methods

### Ethics statement

This study was approved by the Human Subjects Protection Program of the University of California San Diego, La Jolla, California and by the Ethical Boards of Universidad Peruana Cayetano Heredia and Asociación Benéfica PRISMA, Lima, Peru.

### Mosquito collections

**Fig 1:**
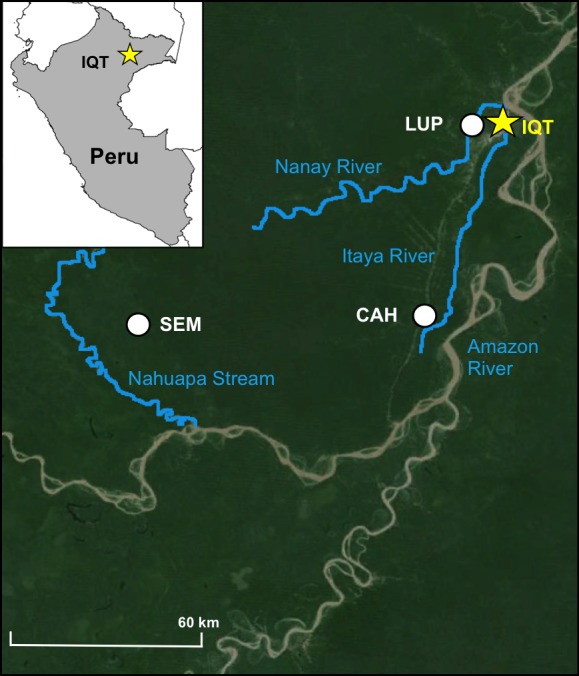
Map of the sites where the mosquito collections were performed in the Department of Loreto, Amazonian Peru.

The strategy of the barrier screen method of collecting mosquitoes outdoors is to intercept and capture mosquitoes transiting between blood feeding and resting sites [20]. Two possible scenarios can be identified: 1) intercepting mosquitoes entering a village seeking a blood-meal after emergence or oviposition; and 2) intercepting blood-fed mosquitoes leaving the village and seeking resting sites for egg development (swamp, creek, stream, forest). In this Peruvian study, barrier screens were placed to intercept mosquitoes flying between house-forest and house-river depending on the specific characteristics of the locality. Mosquito collections were performed in three villages in Loreto Department: Lupuna (LUP) and Cahuide (CAH) in the peri-Iquitos area, and Santa Emilia (SEM), in a remote area ~150 km from Iquitos (Fig 1). Detailed descriptions of these villages are in [15, 22]. In 2013, from March to May, a pilot study was conducted using a single screen in LUP and CAH placed at different points within each village (between the creek/river and village houses). Specimens were collected for 4 nights (6PM- 6AM) each month.

**Fig 2:**
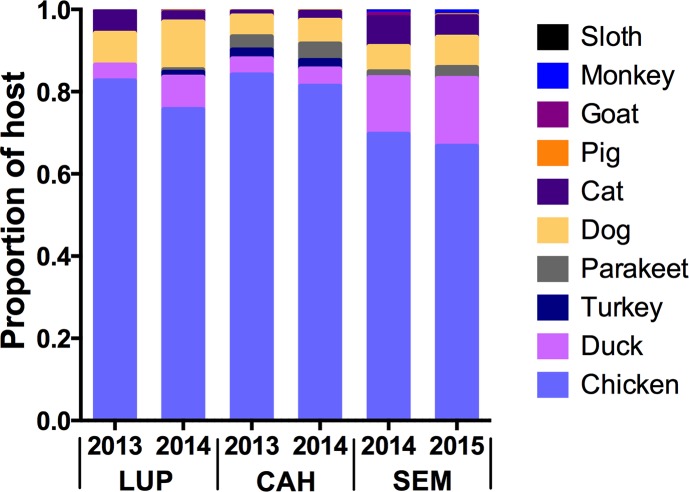
Proportion of the domestic and wild animals in the study localities based on host censuses in 2013–2015. Additional animals seen frequently by the inhabitants were rats, toads, snakes and wild rodents.

Each barrier screen was constructed from a lightweight window screen mesh approximately 15 m long and 2 m high (S1 Fig). Screens were then attached to poles with thin wire. Permission from the inhabitants/owners was obtained prior to any activity, including setting up the barrier screens and performing mosquito collections. Resting mosquitoes from the barrier screens were sampled by manually searching the surface of the screens with a mouth aspirator every hour for 15 minutes on each side, and the location (next to house, forest or river) and height (˃ or ˂ 1m above ground) of mosquitoes was recorded. Mosquitoes were captured and stored by hour of collection and screen side separately. In 2014 (monthly) and 2015 (January-June), the design was slightly modified to include four barrier screens in LUP and CAH to better represent the *An*. *darlingi* population in each locality. When multiple screens were used per village, data from each screen was maintained separately. In SEM, a remote village along the Nahuapa River, collections were performed with two barrier screens for two nights in May-June 2014 and May-September 2015. Additionally, in 2015, daytime mosquito collections (6AM-6PM) with barrier screens were performed two days monthly from January-June in LUP and CAH, and from May-July in SEM. Screen orientation, wind speed and direction were recorded for every collection with a Windmate 300 Wind/Weather Meter. A census questionnaire of domestic hosts present in the study villages was performed in October 2014 in LUP and CAH and May 2015 in SEM (S1 Table, Fig 2). Because the first study was performed a year prior and the animal composition could have changed, the questionnaire included a retrospective question to assess the presence of potential past hosts.

All specimens collected were morphologically identified using entomological keys [23–25] and abdominal status recorded (unfed, blood-fed or gravid). Mosquitoes were stored and labeled individually with silica gel and placed at 4°C until subsequent analysis.

### Estimation of parity and daily survival rate

To estimate the female age composition of the population, in March-April 2014 and February-June 2015 in LUP and CAH a proportion of females were dissected to determine the parity rates per hour, trap and side of trap [26]. Parity is also used as an indicator of mosquito survival under natural conditions. Mosquito longevity (life expectancy) was estimated using Davidson’s methodology (1954) $Age=\frac{1}{logl^{P}}$, where l is the natural logarithm of the constant *P* (daily survival rate). (*P*) was calculated *P* = $\sqrt[{gc}]{PR}$, where *PR* is the ratio of parous mosquitoes and the total number of females dissected, and *gc* is the duration of the gonotrophic cycle in days [27]. A limitation of this calculation is the assumption of accurate estimates of the length of the gonotrophic cycle. We have assumed that two or more blood-meals are required for the first oviposition and that the temporal feeding pattern is not regular, and therefore, we followed the method of calculations proposed by Garret-Jones and Grab [28]. Various studies have estimated the gonotrophic cycle of *An*. *darlingi* to be 2–3 days [29, 30, respectively]. Recently, it was calculated to be 2.19 days in the rainy season and 2.43 in the dry season [31]. Calculations in our study were performed using the 2.19 day estimate based on the timing of our *An*. *darlingi* collections (the rainy season).

### Laboratory procedures

Individual *An*. *darlingi* were bisected between the head/thorax and abdomen and DNA was extracted manually using the DNeasy Blood & Tissue kit (Qiagen). A PCR-RFLP protocol was performed to detect the most common host in the area [32] for all mosquito abdomens in 2013–2015, except for a subsample (60%) of mosquitoes collected in LUP 2014 (due to a extended sample size). In addition, samples were tested for Galliformes (*Gallus gallus* and turkeys; see census and proportion of chickens; Fig 2, S1 Table) following [33], rat and didelphis [34], and monkey [35]. A subsample of the unidentified blood samples was sequenced for the mitochondrial *COI* gene [36] and then compared with sequences in GenBank using BLASTn (http://www.ncbi.nmln.nih.gov) or BOLD SYSTEMS v2.5 (http://www.barcodinglife.org). The best match with identity of 95% or above was recorded.

Detection of *Plasmodium* infection was conducted using real-time PCR of the small subunit of the 18S rRNA, with a triplex TaqMan assay (Life Technologies), as described in [37]. First, DNA was extracted from each specimen of *An*. *darlingi*, then the RT-PCR was conducted on pools of DNA of head/thoraces of five mosquitoes, and finally the pools were analyzed for detection of *P*. *vivax* and *P*. *falciparum*. Specimens from positive pools were tested individually to calculate infection rate (IR).

### Data analysis

HBI was calculated as the proportion of mosquitoes fed on a specific host divided by the number of mosquitoes analyzed (mixed blood-meals were added to totals of each host). To adjust the HBI, mosquitoes with unidentified blood-meals were excluded. This index was calculated monthly in each locality and Chi-square (*χ*^2^) analyses were performed to compare statistical differences temporally and among sites. Host data recorded in the census was used for the calculation of the forage ratio (*w*_*i*_) [38, 39] and selection index (*B*_*i*_) [40], to quantify the preference of mosquitoes for available blood resources. The forage ratio for species *i* was calculated as $wi=\frac{o_{i}}{p_{i}}$, where *o*_*i*_ is the proportion of host species *i* in the blood-meals, and *p*_*i*_ is the proportion of available host in the environment. Forage ratios >1.0 indicate preference and < 1.0 avoidance and selection of another host; ~1.0 means neither preference nor avoidance. The selection index *B*_*i*_ was calculated with the formula $B_{i}=\frac{w_{i}}{\sum_{i=1}^{n}w_{i}}$, where *w*_*i*_ is the forage ratio for species *i* and *n* is the number of blood sources available.

Wind speed was measured at 6:00pm, 12:00am, and 6:00am each collection night in LUP, CAH, and SEM in 2015. For each collection night, mosquito density was aggregated into four 3-hour collection periods (6-9pm, 9pm-12am, 12-3am, and 3-6am). The wind speed at 6:00pm was assigned to the 6-9pm collection time, the wind speed at 12:00am was assigned to the 9pm-12am and 12-3am collection times, and the wind speed at 6:00am was assigned to the 3-6am collection time. The mosquito density was plotted against wind speed for each collection period at each location (n = 48 collection periods each for LUP and CAH, and 40 collection periods for SEM) using the ggplot2 package in RStudio v0.98.1091 [41].

A null-model analysis was used to test whether *An*. *darlingi* feeding habits were random or structured among the three villages, as in [36] and [42]. All specimens with identified blood-meals from 2013–2015 for LUP, 2013–2015 for CAH, and 2014–2015 for SEM were included, and specimens with mixed blood-meals were counted once for each host identified in the blood-meal. We calculated a C-score comparing the blood- meal sources of mosquitoes from the three villages using Ecosim 7.0 and we used the R bipartite package [43] to generate a host-vector quantitative interaction network for the three localities, as in [36].

## Results

### Barrier screen mosquito collections

In 2013, all specimens caught on the screens were collected and identified to determine the potential use of screens for collecting not only Anophelinae but also other Culicidae, potential vectors of parasites and arboviruses. A total of 322 mosquitoes in LUP and 514 in CAH were collected in 6 nights (72 h collection) (Table 1); 94.4% (304/18) of mosquitoes collected in LUP and 89.7% (461/53) of all mosquito species in CAH were females. *Anopheles darlingi* comprised 78.9% and 61.5% of these collections in LUP and CAH, respectively, and *Culex quinquefasciatus* was the second most common species identified in both localities (Table 1). Only one additional species of anopheline, *Anopheles forattini*, was identified (in LUP).

**Table 1:** Number of each mosquito species collected in 2013 pilot survey in LUP and CAH (one barrier screen/locality twice monthly from March to May).

Locality	Species id	*N* (females/males)
**LUP**		
	*Anopheles darlingi*	254 (246/8)
	*Culex quinquefasciatus*	60 (51/9)
	*Mansonia indubitans/titillans*	5 (4/1)
	*Psorophora cingulata*	2 (2/0)
	*Anopheles forattinii*	1 (1/0)
**CAH**		
	*Anopheles darlingi*	316 (304/12)
	*Culex quinquefasciatus*	101 (63/38)
	*Mansonia indubitans/titillans*	72 (72/0)
	*Mansonia humeralis*	15 (15/0)
	*Culex coronator*	6 (3/3)
	*Culex declarator*	1 (1/0)
	*Culex theobaldi*	3 (3/0)

With respect to screen position, in LUP 63.4% of the *An*. *darlingi* were collected on the side facing the houses (In) and 36.6% on the side facing the creek (Out), although this difference was not significant (Kolmogorov-Smirnov test; *p* = 0.4). On both sides of the screen, most of the specimens were collected <1m from the ground (Below; Table 2) (range 76.5–90.2%). In CAH, 61.8% of the mosquitoes were collected on the house side and 38.2% on the creek side, and 93.1% and 84.5% (In and Out, respectively) were caught <1 m from the ground. No differences were found between LUP and CAH for side of the barrier screen. Only 1.62% in LUP and 6.57% in CAH of the *An*. *darlingi* females were determined by visual inspection to be blood-fed, with no differences between screen sides (Table 3).

**Table 2:** Percentage (N) of *Anopheles darlingi* collected above or below 1m on barrier screens in 3 localities by year.

	LUP			CAH			SEM	
Position	2013	2014	2015	2013	2014	2015	2014	2015
**Above (>1m)**	21.2 (40)	23.5 (1,095)	13.2 (135)	9.8 (32)	17.7 (31)	12.7 (30)	16.2 (35)	18.8 (44)
**Below (<1m)**	78.8 (148)	76.5 (3,576)	86.8 (885)	90.2 (295)	82.3 (144)	87.8 (205)	83.8 (181)	81.2 (233)

**Table 3:** Summary of proportion of *An*. *darlingi* visually blood-fed vs. blood-fed determined by molecular analysis, collected using barrier screens in 3 localities from 2013–2015.

			Visually blood-fed mosquitoes			Identified blood-meal			
			In	Out	Total	In	Out	Not id	Total id
Site	Year	*N*	% (*N*)	% (*N*)	% (*N*)	% (*N*)	% (*N*)	% (*N*)	% (*N*)
**LUP**									
**2013**		246	0.81 (2)	0.81 (2)	1.62 (4)	56.8 (138)	35.4 (86)	7.8 (19)	92.2 (243)
**2014**		4,593	0.74 (34)	0.39 (18)	1.13 (52)	55.5 (1,159)	43.8 (914)	0.7 (15)	99.3 (2,084)
**2015**		1,019	6.96 (71)	1.47 (15)	8.43 (86)	50.5 (448)	47 (417)	2.5 (22)	97.5 (887)
**CAH**									
**2013**		330	3.28 (10)	3.28 (10)	6.57 (20)	61.2 (202)	32.7 (108)	6.1 (20)	94 (330)
**2014**		175	6.85 (12)	1.15 (2)	8 (14)	70.8 (119)	17.2 (29)	12 (20)	94 (168)
**2015**		233	9.87 (23)	0.42 (1)	10.3 (24)	57.5 (133)	39.5 (91)	3 (7)	96.5 (231)
**SEM**									
**2014**		216	0.92 (2)	0 (0)	0.92 (2)	70.9 (144)	25.1 (51)	4 (8)	96 (203)
**2015**		277	9.02 (25)	5.41 (15)	14.44 (40)	50.6(137)	47.6 (129)	5 (1.8)	98.1 (271)

Side of screen facing house = In; side of screen facing forest/water = Out.

In 2014, using multiple barrier screens per locality, a total of 4,593 *An*. *darlingi* females were collected in LUP, 175 in CAH and 216 in SEM (Table 2). One specimen of *Anopheles dunhami* in LUP and eighteen *Anopheles benarrochi* B in SEM were also identified as in [14]. In LUP, no significant differences were detected between the sides of four screens tested independently. However, when data were grouped over months there was a significant difference between mosquitoes collected on the side of the houses (In) and creek/vegetation side (Out) (Wilcoxon test; *p* = 0.0313). In CAH, the four barrier screens were not homogeneous, with significant differences in number of mosquitoes collected from each side (K-S; In: *p* = 0.0082 and Out: *p* = 0.0356), and when In/Out were compared by month (K-S; *p* = 0.0022). There were also significant differences between collections in LUP and CAH (K-S, *p* = 0.0336). In SEM, captures in May (two screens) and in June (four screens), were not significantly different between screens.

In 2015, in LUP, 1,019 female mosquitoes were collected, 233 in CAH and 277 in SEM. Most specimens were captured resting < 1m from the ground with little variation among years and sites (Table 2).

**Fig 3:**
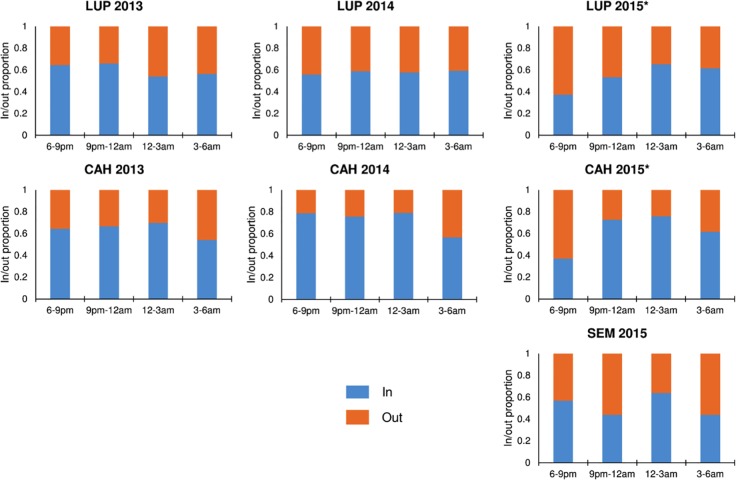
Proportion of *An*. *darlingi* collected on the in (facing house, blue) vs. out (facing forest/water, orange) side of barrier screen by time of collection in LUP, CAH, and SEM, 2013–2015. *Significant difference in the distribution of mosquito collection location by time period (Kruskal-Wallis *p* < 0.0001).

Differences in mosquito density by time of collection and side of barrier screen were tested (Fig 3) with time of collection split into four three-hour periods (6-9pm, 9pm-12am, 12-3am, and 3-6am). In both LUP and CAH in 2015, there was a significant difference in the distribution of mosquito collection location (side of screen) by time period (Kruskal-Wallis *p* < 0.0001 for both sites), with higher proportions of mosquitoes found on the In (facing house) side of the screen from 9pm-12am and 12-3am than from 6-9pm and 3-6am. In LUP and CAH in 2013 and 2014, and in SEM in 2015, there was no significant difference in mosquito density by time of collection (Kruskal-Wallis *p*>0.05).

**Fig 4:**
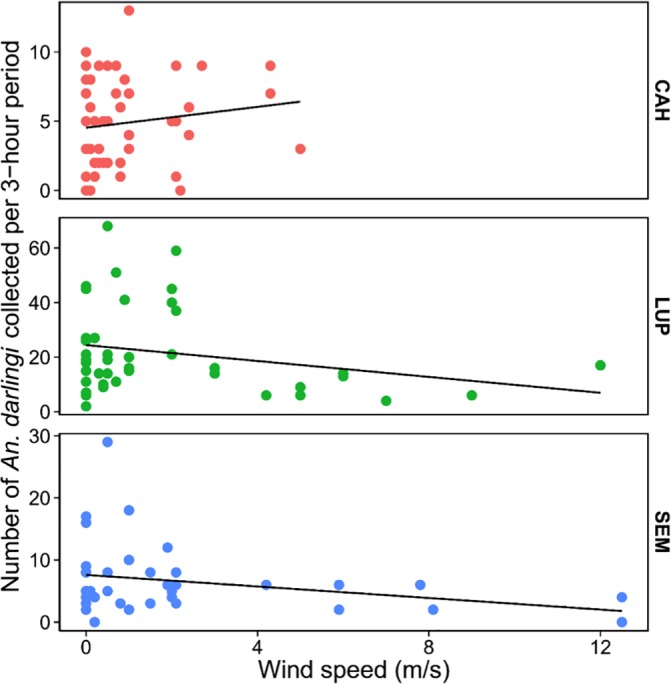
Correlation between density of *An*. *darlingi* on barrier screens and wind speed. Mosquitoes were collected from 6pm-6am from January-June 2015 in CAH and LUP and May-September 2015 in SEM. Linear regression of mosquito density on wind speed shown for each location (CAH: Pearson’s r = 0.14, *p* = 0.34; LUP: Pearson’s r = -0.25, *p* = 0.1; SEM: Pearson’s r = -0.27, *p* = 0.09).

Plots of mosquito density against wind speed for each locality in 2015 are shown in Fig 4. Overall, there was a negative but non-significant correlation between mosquito density and wind speed (Pearson’s r = -0.09, *p* = 0.3). The correlation between mosquito density and wind speed was also negative in LUP (Pearson’s r = -0.25, *p* = 0.1) and SEM (Pearson’s r = -0.27, *p* = 0.09), but was positive in CAH (Pearson’s r = 0.14, *p* = 0.34) (Fig 4).

To investigate the diurnal behavior of *An*. *darlingi*, barrier screen collections were performed in LUP and CAH from January to June, and in SEM from May to June from 6AM to 6PM twice January-June 2015. In LUP a total of 59 *An*. *darlingi* were collected during this period and female activity was reported from 6AM to 9AM and from 2PM to 5PM. In CAH, the number of collected specimens was 23, with an activity similar to LUP. In SEM, 33 mosquitoes were collected, with an extension of the flying activity until 8AM, and beginning again in the evening at 4PM. In LUP, 20.3%, in CAH, 34.8% and in SEM 54.5% of diurnal *An*. *darlingi* specimens were collected on the house side (In).

### Variation in parity and daily survival rate

A total of 583 *An*. *darlingi* females from LUP were dissected in 2014 (12% of the total) and 19 in CAH (11%); in 2015, *n* = 633 in LUP (62%) and *n* = 153 (65%) in CAH were dissected. The monthly mean parity rate in LUP in 2015 was ~ 55% (range 45.6–66.7) and in CAH it was ~ 51% (range 27.8–64.5) (Table 4). No significant differences were found between months or between localities, although in February, the rate was slightly higher compared to June. Mosquito age in LUP in March—April 2014 was 7.47 and 14.21 days, respectively, whereas in 2015 it ranged from 14.21–23.90 days. In CAH, mosquitoes collected in March 2014 were estimated to survive 14.98 days, and between 3.73–20.24 days in 2015 (Table 4).

**Table 4:** Parity rate, daily survival and age of *An*. *darlingi* collected by barrier screens from LUP and CAH, 2014–2015.

		% Nulliparous (*N*)		% Parous (*N*)		% Gravid (*N*)		Daily survival rate (*P*)		Age (days)	
Site/Year		2014	2015	2014	2015	2014	2015	2014	2015	2014	2015
**LUP**	Feb	**-**	10.7 (8)	-	60 (45)	-	29.3 (22)		0.95	-	19.42
	March	25.4 (64)	14.3 (12)	61.5 (155)	66.7 (56)	13.1 (33)	19 (16)	0.94	0.93	7.47	14.21
	April	8.5 (30)	11.9 (13)	37.5 (132)	52.3 (57)	54 (190)	35.8 (39)	0.96	0.94	24.65	17.24
	May	**-**	12.2 (18)	-	52 (77)	-	35.8 (53)		0.94	-	16.89
	June	-	8.8 (19)	-	45.6 (99)	-	45.6 (99)		0.96	-	23.90
**CAH**	Feb	-	12.9 (4)	-	64.5 (20)	-	22.6 (7)		0.94	-	15.85
	March	13.6 (3)	10.2 (4)	50 (11)	64.1 (25)	36.4 (8)	25.7 (10)	0.94	0.95	14.98	20.24
	April	-	17.6 (9)	-	45.1 (23)	-	37.3 (19)		0.92	-	11.28
	May	-	44.4 (8)	-	27.8 (5)	-	27.8 (5)		0.76	-	3.73
	June	-	28.6 (4)	-	57.1 (8)	-	14.3 (2)		0.86	-	6.51

### Blood-meal source identification

**Fig 5:**
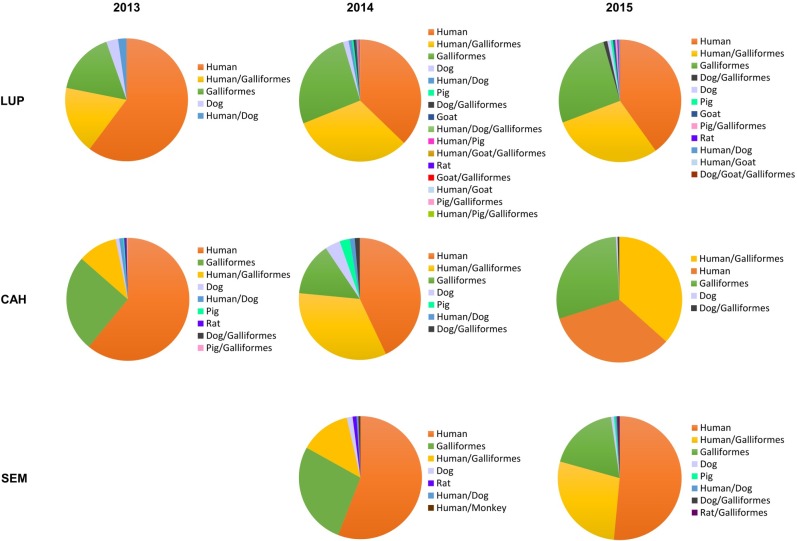
Proportion of blood-meal source, *Anopheles darlingi* collected by barrier screens in LUP, CAH and SEM in 2013–2015.

Blood-meal source was determined for 4,417 *An*. *darlingi* females (S2 Table). A total of 3,214 mosquitoes from LUP, 729 from CAH and 474 from SEM were analyzed. Single-host blood-meals were the highest percentage among the blood-meals detected (69.98%) and human was the most common blood source (42.5%), followed by Galliformes (25.1%) and dog (1.42%; Fig 5). Only 4% of the samples could not be identified to blood-meal source. Multiple blood-meals were found in 1,272 mosquitoes and accounted for 30% of the blood- meals, with 1,262 double feeds in the three localities, and triple feeds (*n* = 10) only identified in LUP.

In total, seventy-three samples with non-identified blood-meal source by PCR-RFLP, were sequenced for 16S ribosomal DNA [36] and mammalian *cytochrome-b* [32]. Only ten were identified as of human origin with the 16S protocol, whereas 23 were consistent with human for *cytochrome-b*.

The distribution of blood-meal source in *An*. *darlingi* presented little temporal or spatial variation. Evaluation of the proportion of feeds on single different hosts showed that in LUP, no significant differences between years were detected by one-way ANOVA analysis; paired Wilcoxon-tests were not significant when comparing years 2013–2014 with 2015 or 2013 and 2014. In CAH, no significant differences between the years 2013–2014, 2014–2015 or among the 3 years were found. In SEM, a non-parametric Mann-Whitney test was not significant comparing 2014 and 2015. For locality comparison, data from the same years and different localities were compared. In 2013, there were no significant differences between LUP and CAH, and in 2014 and 2015 a one-way ANOVA test did not show differences between sites.

HBI was calculated monthly (S3 Table) and annually (Table 5) per locality. In 2013, no significant differences were detected in LUP or CAH. Mean HBI per year was non-significant among localities (LUP, CAH, SEM) and years 2014–2015.

**Table 5:** Summary of variation of *An*. *darlingi* Human Blood Index (HBI) per year and locality.

		HBI				
Year/Locality	LUP (range)	95% CI of mean	CAH (range)	95% CI of mean	SEM (range)	95% CI of mean
**2013**	0.74 (0.71–0.76)	(0.67–0.80)	0.57 (0.46–0.63)	(0.33–0.80)	-	-
**2014**	0.72 (0.66–0.87)	(0.67–0.78)	0.69 (0.63–0.77)	(0.65–0.73)	0.67 (0.66–0.69)	(0.48–0.86)
**2015**	0.65 (0.58–0.7)	(0.61–0.69)	0.67 (0.6–0.77)	(0.6–0.73)	0.79 (0.75–0.82)	(0.69–0.88)

**Fig 6:**
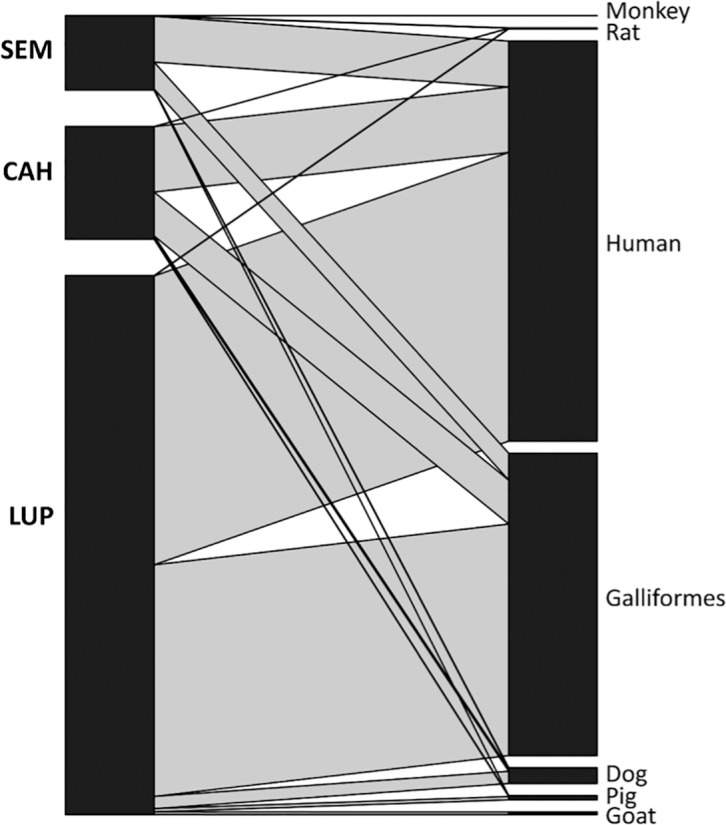
Quantitative interaction network of *An*. *darlingi* blood-meal sources in SEM, CAH, and LUP. Network is based on the analysis of blood-meal source for 4,417 *An*. *darlingi* females collected from 2013–2015.

The Forage Ratio and Host Selection Index were calculated, accounting for single and multiple blood-meals (Table 6). Humans were the preferred source, closely followed by Galliformes, in all three settings for both years. When the Forage Ratio was analyzed, the weight per host was used instead of the numerical presence at the site [36] (S4 Table), Galliformes were by far the preferred host, with humans as the second most favoured. For example in LUP, the Galliforme forage ratio ranged from 10.35 to 17.96 and the human forage ratio from 0.58–0.72. The null model test indicated that the mosquito feeding patterns were aggregated among the localities, indicating that diet overlapped more than expected between the localities, although this finding was only marginally significant (C-score: 0.33, *p* = 0.08). The quantitative interaction network of blood-meal source by locality (Fig 6) supported patterns of organization based on the above-mentioned trophic preferences (humans and Galliformes) from the three mosquito populations (LUP, CAH, SEM).

**Table 6:** Forage ratio (*wi*) and host selection index (*Bi*) of *Anopheles darlingi* in LUP, CAH and SEM from 2013–2015. Values of 1/n of the standardized *wi* or *Bi* indicate no preference, below relative avoidance and >1/n relative preference.

	Collection year			Host abundance		Forage ratio			Selection index			
						(*w*_*i*_)			(*B*_*i*_)			
Site/ Host blood meal	2013	2014	2015	2013	2014	2013	2014	2015	2013	2014		2015
	(*N*)	(*N*)	(*N*)									
**LUP**												
Human	180	1412	602	432	432	1.61	1.24	1.25	0.53		0.13	0.09
Dog	12	61	20	52	77	0.89	0.30	0.23	0.29		0.03	0.01
Galliformes	77	1188	495	557	509	0.53	0.88	0.87	0.17		0.09	0.06
Pig	-	15	10	0	1	-	5.70	9.02	-		0.60	0.69
Goat	-	13	7	4	4	-	1.23	1.57	-		0.13	0.12
**CAH**												
Human	224	116	157	910	910	1.09	0.89	0.79	0.08		0.06	0.31
Dog	7	10	2	35	33	0.89	2.12	0.28	0.06		0.15	0.11
Galliformes	113	73	148	596	478	0.84	1.07	1.43	0.06		0.07	0.57
Pig	2	4	-	4	3	10.58	9.35		0.78		0.69	
**SEM**												
Human	-	137	212	-	212	-	1.36	1.35	-		0.55	0.36
Dog	-	4	4	-	25	-	0.33	0.21	-		0.13	0.05
Galliformes	-	79	125	-	227	-	0.73	0.74	-		0.30	0.20
Pig	-	-	1	-	1	-	-	1.35	-		-	0.36

### *Plasmodium* mosquito infection

A total of 5,387, 362 and 455 mosquitoes in LUP, CAH and SEM, respectively, collected on barrier screens, were tested for *Plasmodium*. The Infection rate (IR) of mosquitoes varied among sites and seasons, ranging from 0.20–3.85 in LUP, 0.51–14.3 in CAH and 0–2.04 in SEM (Table 7). A logistic regression model analysis determined that IR was significantly higher in CAH (*p* = 0.02) and SEM (*p* = 0.003) vs. LUP. No specimens from the diurnal collections in the three localities (*n* = 116) were positive for *P*. *vivax*, independent of the collection season.

**Table 7:** Summary of *Plasmodium* detection in *An*. *darlingi* collected in all localities by barrier screen 2014 and 2015.

Year	Locality	Season^1^	# Collection Months	Total Collected	# inf. *P*. *vivax*	IR
2014	CAH	Rainy	6	157	2	1.27*
2014	CAH	Dry	3	7	1	14.3*
2015	CAH	Rainy	6	198	1	0.51*
2014	LUP	Rainy	6	4356	13^2^	0.30
2014	LUP	Dry	3	26	1	3.85
2015	LUP	Rainy	6	1005	2	0.20
2014	SEM	Rainy	2	196	4	2.04*
2015	SEM	Rainy	2	157	2	1.27*
2015	SEM	Dry	3	102	0	0

^1^Rainy season: Jan-June, dry season: July-Dec

^2^Two *Plasmodium* could not be identified to species.

* Logistic regression, CAH, *P* = 0.02; SEM, *P* = 0.003.

## Discussion

Ours is the first study to conclusively demonstrate that *An*. *darlingi* readily feeds on Galliformes. Overall, the feeding preference of *An*. *darlingi* in the Peruvian Amazon is more variable than previous studies have assumed. In addition, a consistent pattern of blood-meal source was observed at each site every year of collection: mosquitoes feeding only on humans, only on chickens, or on both hosts. This consistency could suggest the co-occurrence of different subpopulations within a metapopulation, with local adaptation as the main driving force.

A single metapopulation was initially detected in *An*. *darlingi* in the Iquitos area with AFLPs [44] and microsatellite markers [45]. However, using 2x the number of microsatellites, a population replacement event was detected between 2006 and 2012 and two subpopulations were detected, one significantly more prevalent in highway compared with riverine habitat [20]. This recent genetic structure could explain some of the heterogeneity in feeding preferences of *An*. *darlingi* among localities [45, 46]. Additional studies, focused on intrinsic host preference, vector density and social practices of the human population might elucidate the basis for the described behavior and whether some *An*. *darlingi* populations are under selective pressure for host preference or whether this pattern is strongly correlated with host availability.

Similar HBI across the dry and rainy seasons and between populations infers that mosquitoes maintain their host preference behavior independent of local ecological conditions. In an earlier investigation of HBI of *An*. *darlingi* in riverine villages in Amapá State, Brazil [18], researchers reported high among-village variance (HBI 0.131–0.435) and ~10% of mixed blood-meals overall, mainly from cattle and pigs. In contrast, in our study, there was virtually no variance in HBI among localities, HBIs were higher (0.58–0.79) and ~30% of blood-meals were mixed, with Galliformes as the primary alternate host. Because HBI is an integral parameter of the vectorial capacity formula (the daily rate of malaria transmission from a single infected human, assuming every bite from an infected mosquito leads to transmission) [2], our data suggest that *An*. *darlingi* is a more effective vector in the peri-Iquitos area compared with Amapá state, Brazil. Curiously, in Tanzania, *An*. *arabiensis* avoids, and may be repelled by, the volatiles of chickens [47]. Subgenera *Nyssorhynchus* (*An*. *darlingi)* and *Cellia* (*An*. *arabiensis)* were estimated to have diverged ~94 million years ago [48]; therefore their olfactory responses are expected to have evolved differentially.

The present study provides evidence of the successful use of barrier screens to collect blood-fed *An*. *darlingi* mosquitoes in Amazonian Peru. Initially, in 2013, we conducted preliminary barrier screen collections with Procopack aspirators in LUP and CAH from 5 to 8 AM for 6 days/collection in March-May in at least 10 houses each time, but only one *An*. *darlingi* specimen was caught. Interestingly, in Iquitos the Procopack effectively collected indoor resting Culicidae including *Aedes aegypti* and *Culex pipiens* complex [49]. One explanation for our failure to find *An*. *darlingi* using the Procopack despite extensive searching could be due to its singular resting and biting behavior in this region.

*Anopheles darlingi* resting behavior varies across its range [50]: in Venezuela, Guyana [51] and in Brazil, in Matto Grosso and in southern Amazonas [52, 53] it rests during the day inside houses (endophily). In contrast, in Suriname, using exit traps, a peak departure from the dwelling was observed at sunrise [54] and in Brazil *An*. *darlingi* was resting indoors only at night [55]. In Amapá state, Brazil, resting mosquitoes were collected after sunrise (6AM-7AM) under houses and in peridomestic vegetation [18]. In French Guiana, no resting *An*. *darlingi* were collected indoors after pyrethroid spray, from pit-shelters or in the shade in the peridomestic area [56]. In our study, overall differences detected between screen sides may reflect the relative nearness of screens to houses, resulting in the interception of a higher proportion of blood fed *An*. *darlingi* leaving the peridomestic area, compared with questing females, entering the village from numerous resting and/or breeding sites. In CAH, we hypothesize that additional differences among screens and between months could result from a much smaller population of *An*. *darlingi* intercepted in this village. Our results constitute a major accomplishment: the use of barrier screens in this setting to overcome the difficulty of performing host-independent sampling for determining blood-meal sources.

The success of individual mosquito blood-meal identification in this study (range of 92.2–99.3%), was remarkably high when compared to visually blood-fed mosquitoes (0.92%-14.44%). When analysis is restricted only to the latter, information from partial blood-meals or partially digested blood is missed, leading to underestimation of the proportion of host sources (up to 18.7%); hence, a miscalculation of HBI [57]. One limitation of our study was the lack of identification of potential wild animal hosts; use of novel targeted high-throughput sequencing [58] would rectify this.

In LUP, the age of the mosquito population at each time point is enough to sustain the sporogonic cycle of *P*. *vivax* (range 7.24–9.13 days; calculated by the Moshkovsky method in [31]), whereas in CAH the population is, in general, younger, but with non-dangerously aged mosquitoes only in May and June. The proportion of young females might be explained by differential dispersal and aggregation of different age classes of *An*. *darlingi* populations, as previously reported for *An*. *farauti* in Papua New Guinea [59]. Use of 2.19 days of the gonotrophic cycle [31] could have produced a miscalculation in the age parameter. For instance, gravid females may experience delays while searching for suitable oviposition sites or there could be variation in extrinsic environmental conditions within this population of *An*. *darlingi* [60]. Because of the natural development of the parasite within the mosquito, a longer life-span is related to a higher potential to transmit malaria [61]. Parity is also associated with seasonality, i.e., mosquitoes generally survive longer during the rainy season [62,63], but see [64].

Overall, our study provides unreported information of the blood-meal preferences of *An*. *darlingi* in the peri-Iquitos area, which will be the base-line to compare potential changes in the behavior of these mosquito populations. HBI, together with other malaria metrics such as HBR or EIR, should be taken into consideration for surveillance and epidemiological studies of malaria transmission.

## Supporting information

S1 Fig **Construction and set up of the barrier screens in Iquitos, Peru;** (A, B, C, F): 2 m high and 15m long. Screens were examined hourly by flashlight and resting mosquitoes captured by aspiration (D, E).
(TIF)

S1 Table Census of domestic and wild animals in the study localities 2013–2015. Rats, toads, snakes and wild rodents were other animals frequently observed by the inhabitants.
(DOCX)

S2 Table Summary of *An*. *darlingi* blood-meal sources per year per locality. (DOCX)

S3 Table Monthly variation of Human Blood Index (HBI) for *An*. *darlingi* in three sites. (DOCX)

S4 Table Forage ratio (FR) of *An*. *darlingi* using host biomass. Mean weight of hosts was: human (65kg), dog (25 kg), chicken (1.5kg), turkey (13.1 kg), pig (90kg), goat (45 kg).
(DOCX)

S1 Dataset Summary of mosquito collections by barrier screens methodology. Collection site and dates of collection, mosquito species identification and side and height of the barrier screens are designated for each mosquito used in the analysis.
(XLSX)

S2 Dataset Mosquito blood-meals. The file shows, for each mosquito analyzed, the source of blood-meal identified.
(XLSX)
