# Supplementary figures and images for: Intensive trapping of blood-fed Anopheles darlingi in Amazonian Peru reveals unexpectedly high proportions of avian blood-meals
Source: PLoS Negl Trop Dis. 2017 Feb 23;11(2):e0005337. doi: 10.1371/journal.pntd.0005337 (PMC5322880; doi:10.1371/journal.pntd.0005337)

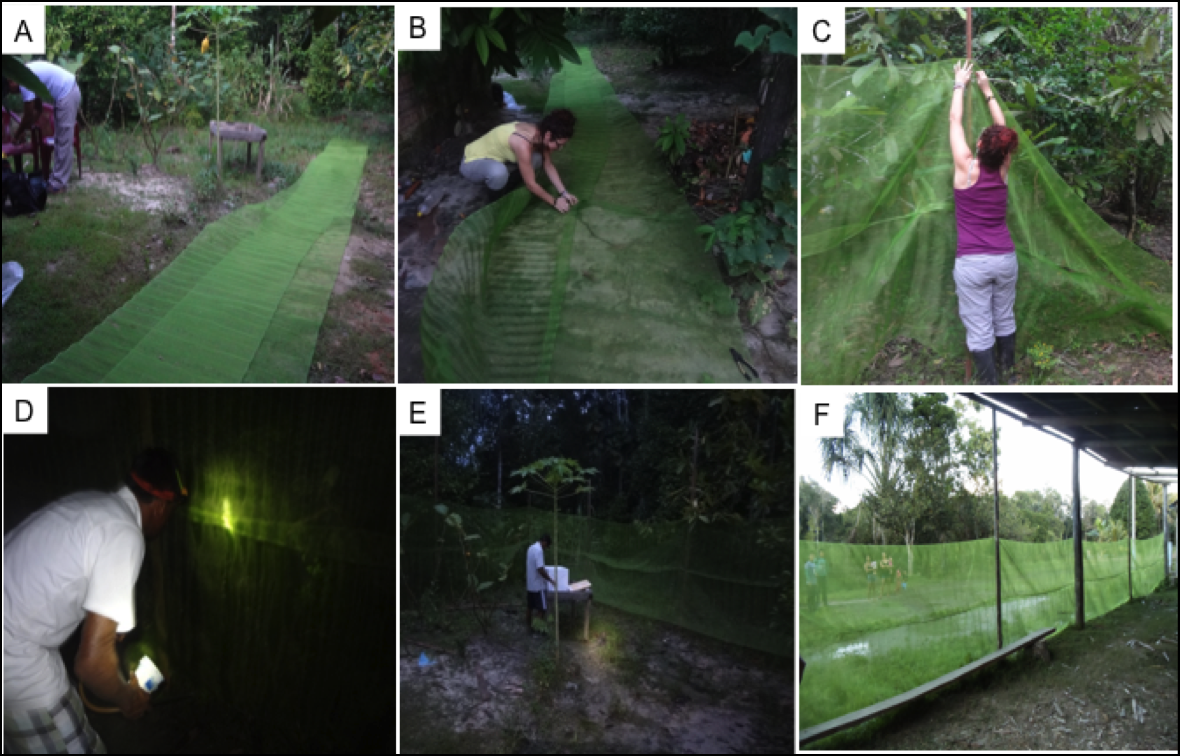

Supplement: S1 Fig — Construction and set up of the barrier screens in Iquitos, Peru; (A, B, C, F): 2 m high and 15m long. Screens were examined hourly by flashlight and resting mosquitoes captured by aspiration (D, E). (TIF) [file pntd.0005337.s001.tif]
