# Supplementary material for: Intensive trapping of blood-fed Anopheles darlingi in Amazonian Peru reveals unexpectedly high proportions of avian blood-meals
Source: PLoS Negl Trop Dis. 2017 Feb 23;11(2):e0005337. doi: 10.1371/journal.pntd.0005337 (PMC5322880; doi:10.1371/journal.pntd.0005337)
Supplement: S1 Table — Rats, toads, snakes and wild rodents were other animals frequently observed by the inhabitants. (DOCX) [file pntd.0005337.s002.docx]

**S1 Table.** **Census of domestic and wild animals in the study localities 2013, 2014 and 2015.** **Rats, toads, snakes and feral rodents were other animals frequently observed by the inhabitants.**

| Site/Year | **2013** | **2014** | **2015** |
| --- | --- | --- | --- |
| **LUP** |  |  |  |
| Chicken (D) | 557 | 501 | - |
| Duck (D) | 26 | 52 | - |
| Turkey (D) | 0 | 8 | - |
| Parakeet (W) | 0 | 4 | - |
| Dog (D) | 52 | 77 | - |
| Cat (D) | 35 | 15 | - |
| Pig (D) | 0 | 1 | - |
| Goat (D) | 4 | 4 | - |
| **CAH** |  |  |  |
| Chicken (D) | 581 | 466 | - |
| Duck (D) | 27 | 24 | - |
| Turkey (D) | 15 | 12 | - |
| Parakeet (W) | 22 | 23 | - |
| Dog (D) | 35 | 33 | - |
| Cat (D) | 7 | 11 | - |
| Pig (D) | 4 | 3 | - |
| Monkey (W) | 0 | 1 | - |
| **SEM** |  |  |  |
| Chicken (D) | - | 248 | 227 |
| Duck (D) | - | 49 | 56 |
| Parakeet (W) | - | 5 | 9 |
| Dog (D) | - | 22 | 25 |
| Cat (D) | - | 25 | 17 |
| Pig (D) | - | 0 | 1 |
| Goat (D) | - | 3 | 0 |
| Monkey | - | 2 | 5 |
| Sloth (W) | - | 2 | 0 |

D: Domestic; W: Wild.
