# Supplementary material for: Intensive trapping of blood-fed Anopheles darlingi in Amazonian Peru reveals unexpectedly high proportions of avian blood-meals
Source: PLoS Negl Trop Dis. 2017 Feb 23;11(2):e0005337. doi: 10.1371/journal.pntd.0005337 (PMC5322880; doi:10.1371/journal.pntd.0005337)
Supplement: S2 Table — (DOCX) [file pntd.0005337.s003.docx]

**S2 Table. Summary of *An. darlingi* blood meal sources per year per locality.**

|  | **LUP** | | | **CAH** | | | **SEM** | |
| --- | --- | --- | --- | --- | --- | --- | --- | --- |
| **Host blood meal/Year collection** | **2013**  (*N*) | **2014**  (*N*) | **2015**  (*N*) | **2013**  (*N*) | **2014**  (*N*) | **2015**  (*N*) | **2014**  (*N*) | **2015**  (*N*) |
| *Single blood meals* |  |  |  |  |  |  |  |  |
| Human | 135 | 746 | 347 | 189 | 64 | 75 | 109 | 137 |
| Dog | 7 | 30 | 8 | 3 | 6 | 1 | 3 | 2 |
| Galliformes | 37 | 532 | 228 | 79 | 21 | 65 | 53 | 49 |
| Pig | - | 9 | 5 | 1 | 4 | - | - | 1 |
| Goat | - | 7 | 5 | - | - | - | - | - |
| Rat | - | 2 | 2 | 1 | - | - | 2 | - |
| *Mixed blood meals* |  |  |  |  |  |  |  |  |
| Human/Dog | 5 | 17 | 2 | 3 | 2 | - | 1 | 1 |
| Human/Galliformes | 40 | 635 | 252 | 32 | 50 | 82 | 26 | 74 |
| Human/Pig | - | 4 | - | - | - | - | - | - |
| Human/Goat | - | 1 | 1 | - | - | - | - | - |
| Human/Monkey | - | - | - | - | - | - | 1 | - |
| Dog/Galliformes | - | 9 | 9 | 1 | 2 | 1 | - | 1 |
| Pig/Galliformes | - | 1 | 5 | 1 | - | - | - | - |
| Goat/Galliformes | - | 2 | - | - | - | - | - | - |
| Rat/Galliformes | - | - | - | - | - | - | - | 1 |
| Human/Dog/Galliformes | - | 5 | - | - | - | - | - | - |
| Human/Goat/Galliformes | - | 3 | - | - | - | - | - | - |
| Human/Pig/Galliformes | - | 1 | - | - | - | - | - | - |
| Dog/Goat/Galliformes | - | - | 1 | - | - | - | - | - |
| Not identified | 19 | 80 | 22 | 20 | 19 | 7 | 8 | 5 |
| All samples tested | 243 | 2,084 | 887 | 330 | 168 | 231 | 203 | 271 |
