# Supplementary material for: Intensive trapping of blood-fed Anopheles darlingi in Amazonian Peru reveals unexpectedly high proportions of avian blood-meals
Source: PLoS Negl Trop Dis. 2017 Feb 23;11(2):e0005337. doi: 10.1371/journal.pntd.0005337 (PMC5322880; doi:10.1371/journal.pntd.0005337)
Supplement: S3 Table — (DOCX) [file pntd.0005337.s004.docx]

**S3 Table. Monthly variation of Human Blood Index (HBI) for *An. darlingi* in three sites.**

|  | **2013** | | | **2014** | | | | | | | | | **2015** | | | | | | | | |
| --- | --- | --- | --- | --- | --- | --- | --- | --- | --- | --- | --- | --- | --- | --- | --- | --- | --- | --- | --- | --- | --- |
|  | **M** | **A** | **M** | **J** | **F** | **M** | **A** | **M** | **J** | **A** | **O** | **D** | **J** | **F** | **M** | **A** | **M** | **J** | **J** | **A** | **S** |
| **HBI LUP** | 0.75 | 0.71 | 0.76 | 0.67 | 0.72 | 0.81 | 0.66 | 0.68 | 0.71 | 0.76 | 0.87 | 0.66 | 0.68 | 0.58 | 0.63 | 0.66 | 0.66 | 0.70 | - | - | - |
| **HBI CAH** | 0.63 | 0.71 | 0.67 | 0.71 | 0.66 | 0.66 | 0.63 | 0.72 | 0.77 | 0.71 | - | - | 0.65 | 0.64 | 0.6 | 0.77 | 0.65 | 0.72 | - | - | - |
| **HBI SEM** | - | - | - | - | - | - | - | 0.66 | 0.69 | - | - | - | - | - | - | - | 0.75 | 0.82 | 0.77 | 0.81 | 0.79 |
