# Supplementary material for: Intensive trapping of blood-fed Anopheles darlingi in Amazonian Peru reveals unexpectedly high proportions of avian blood-meals
Source: PLoS Negl Trop Dis. 2017 Feb 23;11(2):e0005337. doi: 10.1371/journal.pntd.0005337 (PMC5322880; doi:10.1371/journal.pntd.0005337)
Supplement: S4 Table — Mean weight of hosts was: human (65kg), dog (25 kg), chicken (1.5kg), turkey (13.1 kg), pig (90kg), goat (45 kg). (DOCX) [file pntd.0005337.s005.docx]

**S4 Table. Forage ratio (FR) of *An. darlingi* using host biomass.** Mean weight of hosts was: human (65kg), dog (25 kg), chicken (1.5kg), turkey (13.1 kg), pig (90kg), goat (45 kg).

|  | **Host** | **FR** |
| --- | --- | --- |
| **LUP 2013** |  |  |
|  | *Human* | 0.72 |
|  | *Dog* | 1.03 |
|  | *Galliformes* | 10.35 |
| **LUP 2014** | *Human* | 0.58 |
|  | *Dog* | 0.36 |
|  | *Galliformes* | 16.06 |
|  | *Pig* | 1.92 |
|  | *Goat* | 0.83 |
| **LUP 2015** | *Human* | 0.58 |
|  | *Dog* | 0.28 |
|  | *Galliformes* | 15.86 |
|  | *Pig* | 3.04 |
|  | *Goat* | 1.06 |
| **CAH 2013** | *Human* | 0.67 |
|  | *Dog* | 1.42 |
|  | *Galliformes* | 18.79 |
|  | *Pig* | 0.98 |
| **CAH 2014** | *Human* | 0.59 |
|  | *Dog* | 3.64 |
|  | *Galliformes* | 25.66 |
|  | *Pig* | 4.45 |
| **CAH 2015** | *Human* | 0.52 |
|  | *Dog* | 0.48 |
|  | *Galliformes* | 34.25 |
| **SEM 2014** | *Human* | 0.66 |
|  | *Dog* | 0.42 |
|  | *Galliformes* | 15.55 |
| **SEM 2015** | *Human* | 0.66 |
|  | *Dog* | 0.27 |
|  | *Galliformes* | 15.92 |
|  | *Pig* | 0.48 |
